# Are the rapid tranquilisation nice guidelines adhered to, in patients with agitated/aggressive behavior? a QIP

**DOI:** 10.1192/bjo.2021.499

**Published:** 2021-06-18

**Authors:** Asha Dhandapani, Sathyan Soundararajan, Rajvinder Sambhi

**Affiliations:** BCUHB

## Abstract

**Aims:**

To explore whether the NICE guidelines for rapid tranquilisation are adhered to in the Psychiatric intensive care unit (PICU/ Tryweryn).

**Method:**

Data were collected by core trainees. Standards were taken from NICE guidelines NG10. All patients who had received rapid tranquilisation, that were in PICU from August 2019 to February 2020 were considered in this a.

**Result:**

During the first PDSA, we discussed with the staff in the ward regarding the protocol. Prior to actually starting the second audit, the adherence was noted to be low. However following persistence and having created a protocol jointly with the ward manager, we could see the difference. The staff were appreciated for their efforts in maintaining 100% adherence. The same was intended to be continued with some positive reinforcement from the auditing team. Over the first 2 months, 12 patients received Rapid Tranquilisation. Out of these 12, we randomly selected 4 patients to find the adherence of the NICE guidelines to be 100 per cent. The predictions regarding the adherence to protocol showed that the PDSA was successful.

During the second PDSA, the adherence was 100% again. The adherence to the protocol has been followed for not just the sample that was selected, but for the entire set of patients who received the Rapid Tranquilisation. Following this QIP, we formatted the proforma which included the services to be provided/ actions to be taken, Post Rapid Tranquilisation physical health monitoring and response to medication.

**Conclusion:**

The utilisation of de-escalation techniques and behavioural support plans that was person-centred in turn brought down the rate of Rapid Tranquilisation successfully. Thus placing our PICU as having the least restraints in the UK in 2019 (Second least 3/ month). Our PICU was awarded the prestigious Nursing Times Team of the Year Award for their pioneering work.

Following this QIP, we then formatted the proforma for Rapid Tranquilisation which included the services to be provided/ actions to be taken, Post Rapid Tranquilisation physical health monitoring and patients response to medication. The PICU will continue to maintain this 100% standard and we would then consider extending the Audit to both Open wards and PICU in entire North Wales.

